# A Case of Progressive Bulbar Paralysis

**Published:** 1877-07

**Authors:** S. W. Seldner


A Case of Progressive Bulbar Paralysis.—*By S. W. Seldner, M.D.*—C. R., aged 23, barber, was admitted into the Hebrew Hospital on the 12th of February, but did not come under my direct care until the first day of March, at which time I assumed charge of the institution. The following history was obtained from him: Patient has never had any bodily infirmities that confined him to his bed, prior to the present attack; has never had any rheumatic or gouty affections; nor has he had any venereal disease, save a gonorrhœa which readily yielded to treatment.

About fourteen months ago he had an attack of angina tonsillaris, for which he was treated. The first symptoms which attracted his attention to his present complaint, occurred about one year ago, at which time he was subject to severe pain in the head, occurring paroxysmally, and principally localized in the occipital region, and extending from there down the spinal column to the junction formed by the cervical and dorsal vertebræ.

There was also more or less vertigo, and in addition to this the patient noticed after the lapse of a few weeks a peculiar weakness in articulating; certain letters and syllables were difficult to pronunciate, and when pronounced were not always intelligible to those to whom his remarks were addressed; in fact, the condition known as "alalie" was what we had in the particular case.

It was not long before symptoms of dysphagia developed themselves, and deglutition was so much interfered with that at times the food, especially the fluid portions, would cause him to eject a portion, or it would produce suffocative symptoms of an alarming character. This was more particularly present a day prior to his death. With all this difficulty there was a remarkably good appetite, and the patient never thought of returning any of the food that had been sent him, even if it did not tickle his palate.

His sense of taste was not wholly interfered with. If quinine was placed upon his tongue, it required an interval of about five minutes to ascertain its presence through its bitter taste; there was a continual dribbling of saliva, and during sleep this symptom annoyed him very much.

The most distressing symptom (to him) was the gradually developing weakness in his upper extremities, and more particularly the right, which was very much wasted, so much so that when he apposed the thumb to the index finger, the prominence formed by the ball of the thumb was entirely wanting; the deltoids, biceps, triceps, and pectoral muscles were atrophied so that but a few fibres remained where once these muscles existed.

Tactile sensibility and electro-muscular excitability were much diminished; in fact, his upper extremities were completely anæsthetic.

A pin or needle introduced to the depth of five or six lines was not perceived, and unless he saw his hands held he was not aware that any one was holding them. Under these circumstances it is evident that the patient was so helpless that he could not use his upper extremities, even for the purpose of supplying himself with food; and in this condition he was compelled to rely on the commiseration of his faithful nurse for sustenance.

There were no symptoms referable to the organs of vision or hearing, nor was there any circulatory disturbance.

His digestion was good and his bowels were regular. His respiratory organs, which from the physical examination failed to reveal a diseased condition, caused him considerable suffering; he would suddenly have attacks, which, to use his own words, seemed to him as "if he could never breath again," and these would occur diurnally and nocturnally.

An examination of his urine for albumen and sugar gave a negative result.

In this way he continued to grow (physically) gradually less, until the 29th day of March, when he suddenly expired, after previously having partaken of a hearty meal and some brandy which a fellow-sufferer had contributed.

The parents of the young man would under no consideration consent to a post-mortem examination; therefore we are compelled to rely on the ante-mortem investigation as to the correctness of our diagnosis. (Prof. Arnold, who saw the patient in consultation, agreed with me in the diagnosis.) There is no disease with which it might be confounded, yet the similarity of some of the symptoms to those produced by other lesions should make us careful in order to trace the trouble to its proper seat.

The tottering gait might cause one to confound it with progressive locomotor ataxy; yet the patient could retain the erect posture with his eyes firmly closed, and the lancinating pains through the knees, so characteristic of this malady, were altogether wanting.

From sclerosis it is distinguished through the absence of nystagmus and tremors during intended movements. In fact, when the medullæ spinalis is affected, as was the case with our patient (judging from the atrophy of the muscles), there is always, or generally, a condition of myelitis tending to sclerosis.

In a given case it is difficult to say which is the original affection, and some writers content themselves by giving that priority which first manifested itself; thus if the gradual wasting of the muscles was the first appreciable symptom the patient observed, independent of the gradually developing difficulty in swallowing, they call it progressive muscular atrophy, and vice versa. Practically speaking, it makes little difference, for the two diseases are so intimately associated, or rather blended, that their recognition as being one or the other is a matter of small importance.

Even their pathological conditions are so nearly analogous that it is difficult at times to draw these fine distinctions, and if we believe with some of the most recent writers on this particular subject, we can content ourselves that if progressive bulbar paralysis is accompanied with the symptoms of progressive muscular atrophy, we may rest assured that pathological changes have taken place of an atrophic degenerative character in the medullæ oblongata and medullæ spinalis.

The disease being always of central origin.

In reference to treatment (so far as our literature is concerned), it leaves much to be wished for, and from the tendency of the malady but little can be done.

The preparations of silver, gold, strychnia, phosphorus, and zinc have each had their admirers; bromide of potassium has been tried and abandoned; electricity has been extensively used, and although it does not prevent the fatal issue, yet for a time during its use the symptoms either remain stationary, or temporary improvement takes place.

When the case has advanced so far that the patient is unaable to swallow, it becomes necessary for us to sustain life through the introduction of food by means of the œsophageal tube.—*Maryland Med. Jour.*

